# Hirshfeld surface analysis and crystal structure of *N*-(2-meth­oxy­phen­yl)acetamide

**DOI:** 10.1107/S2056989019006972

**Published:** 2019-05-21

**Authors:** Mavise Yaman, Necmi Dege, Mzgin M. Ayoob, Awaz J. Hussein, Mohammed K. Samad, Igor O. Fritsky

**Affiliations:** a Ondokuz Mayıs University, Faculty of Arts and Sciences, Department of Physics, 55139, Samsun, Turkey; b Department of Chemistry, College of Education, Salahaddin University-Erbil, Erbil-Kurdistan, Iraq, 44002; c Department of Chemistry, College of Education, Salahaddin University – Hawler, Erbil-Kurdistan, Iraq; d Taras Shevchenko National University of Kyiv, Department of Chemistry, 64, Vladimirska Str., Kiev 01601, Ukraine

**Keywords:** crystal structure, hydrogen bonding, amide, meth­oxy­phen­yl, Hirshfeld surface analysis

## Abstract

The title compound, C_9_H_11_NO_2_, was obtained as unexpected product from the reaction of (4-{2-benz­yloxy-5-[(*E*)-(3-chloro-4-methyl­phen­yl)diazen­yl]benzyl­idene}-2-phenyl­oxazol-5(4*H*)-one) with 2-meth­oxy­aniline in the presence of acetic acid as solvent. The amide group is not coplanar with the benzene ring, as shown by the C—N—C—O and C—N—C—C torsion angles of −2.5 (3) and 176.54 (19)°, respectively. Hirshfeld surface analysis and two-dimensional fingerprint plots indicate that the most important contributions to the crystal packing are from H⋯H (53.9%), C⋯H/H⋯C (21.4%), O⋯H/H⋯O (21.4%) and N⋯H/H⋯N (1.7%) inter­actions.

## Abstract 2

The title compound, C_9_H_11_NO_2_, was synthesized and characterized in the solid state. The mol­ecular Hirshfeld surfaces were obtained to determine the inter­actions between the mol­ecules and explore the nature of the packing of the mol­ecules in the crystal.

## Chemical context   

The amide function is one of the most important linkages in natural chemistry. It is the key linker in peptides and a number of polymers, and is additionally found in numerous pharmaceuticals and other items (Dam *et al.*, 2010 ▸) with natural activity, including about 25% of commercially available drugs. Consequentially, the amide bond is a standout amongst the most vital changes in a current natural blend (Ojeda-Porras & Gamba-Sánchez, 2016 ▸). In the light of such discoveries, we report the crystal structure of the title compound.
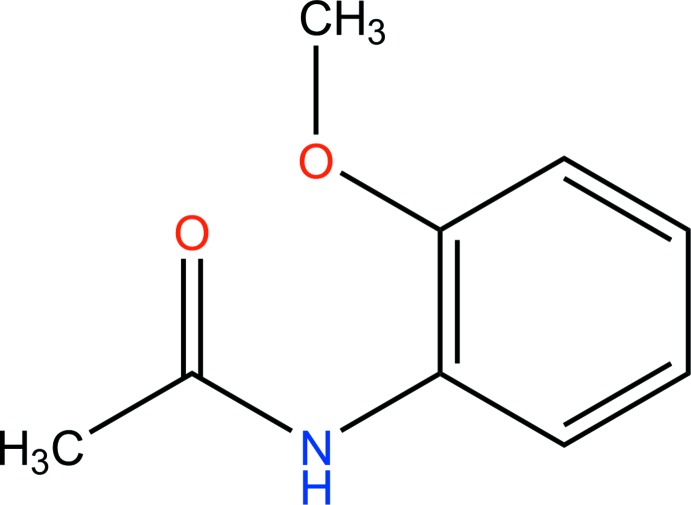


## Structural commentary   

**Figure 1:**
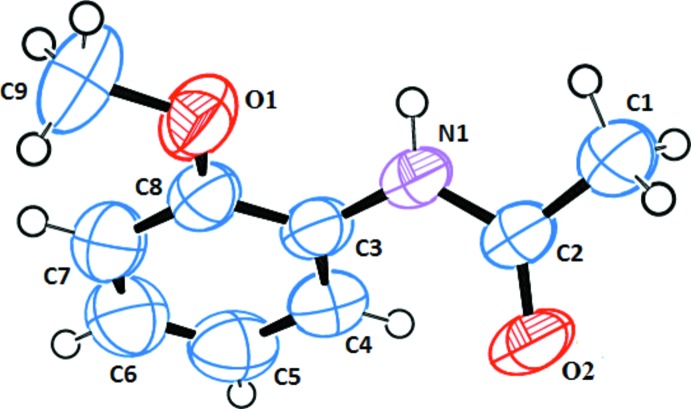
The asymmetric unit of the title compound with displacement ellipsoids drawn at the 50% probability level.

The mol­ecular structure of the asymmetric unit of the C_9_H_11_NO_2_ compound is shown in Fig. 1 ▸. The N1—C2, C2—O2 and C2—C1 bond lengths are 1.347 (2), 1.2285 (19) and 1.480 (3) Å, respectively. The C2—O2 bond in the amide group shows partial double-bond character and is similar in length to those found in amide compounds in the literature [1.215 (2) Å (Kansiz *et al.*, 2018 ▸), 1.240 (2) Å (Aydemir *et al.*, 2018 ▸) and 1.2205 (10) Å (Chkirate *et al.*, 2019 ▸)]. The C3—C8 benzene ring is planar with an r.m.s. deviation of 0.0019. The amide group is not coplanar with the benzene ring, as shown by the C3—N1—C2—O2 and C3—N1—C2—C1 torsion angles of −2.5 (3) and 176.54 (19)°, respectively.

## Supra­molecular features   

**Figure 2:**
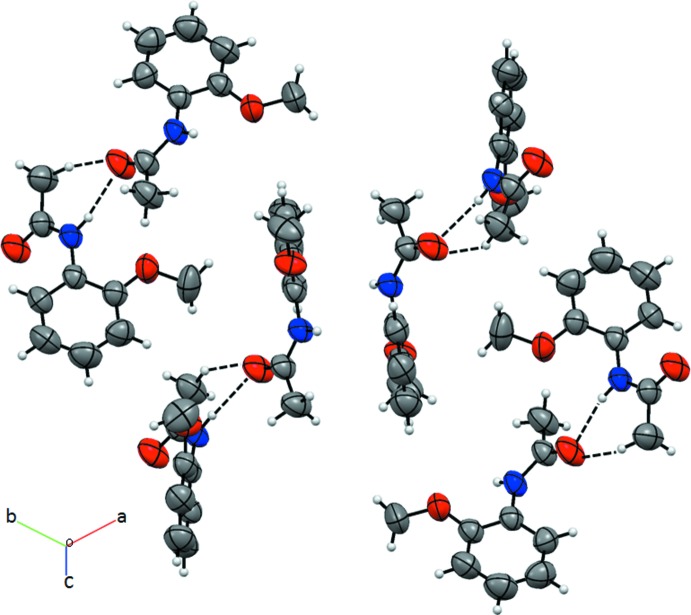
A partial view of the crystal packing. Dashed lines denote the inter­molecular C—H⋯O and N—H⋯O hydrogen bonds (Table 1 ▸).

In the crystal, adjacent mol­ecules are linked by weak C—H⋯O hydrogen bonds, forming supra­molecular chains propagating along the *a*-axis direction (Table 1 ▸ and Fig. 2 ▸). The chains are further connected by weak C—H⋯π inter­actions.

## Hirshfeld surface analysis   

**Figure 3:**
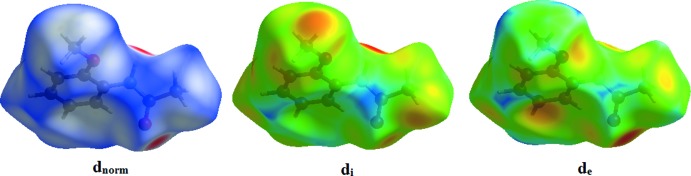
The Hirshfeld surface of the title compound mapped over *d*_norm_, *d*_i_ and *d*_e_.

**Figure 4:**
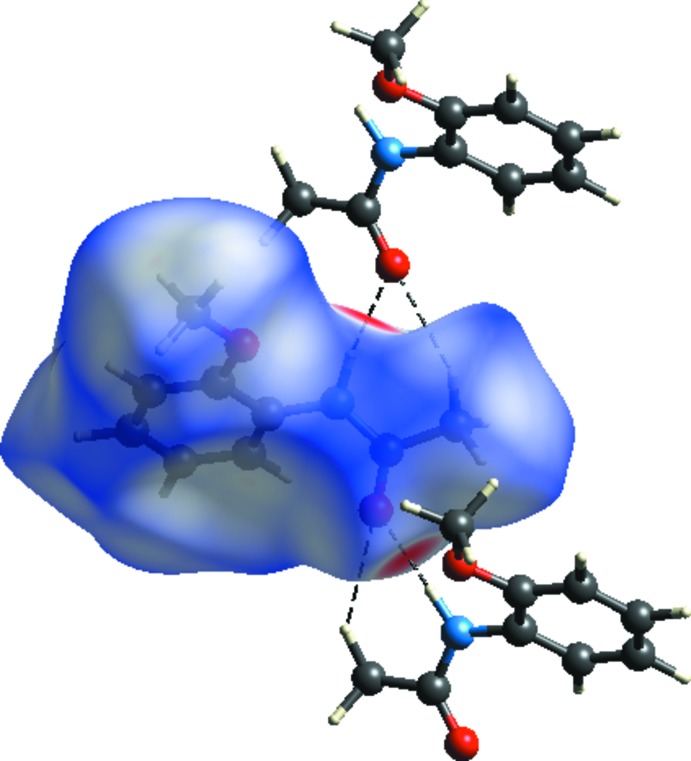
*d_norm_* mapped on the Hirshfeld surface for visualizing the inter­molecular inter­actions of the title compound.

**Figure 5:**
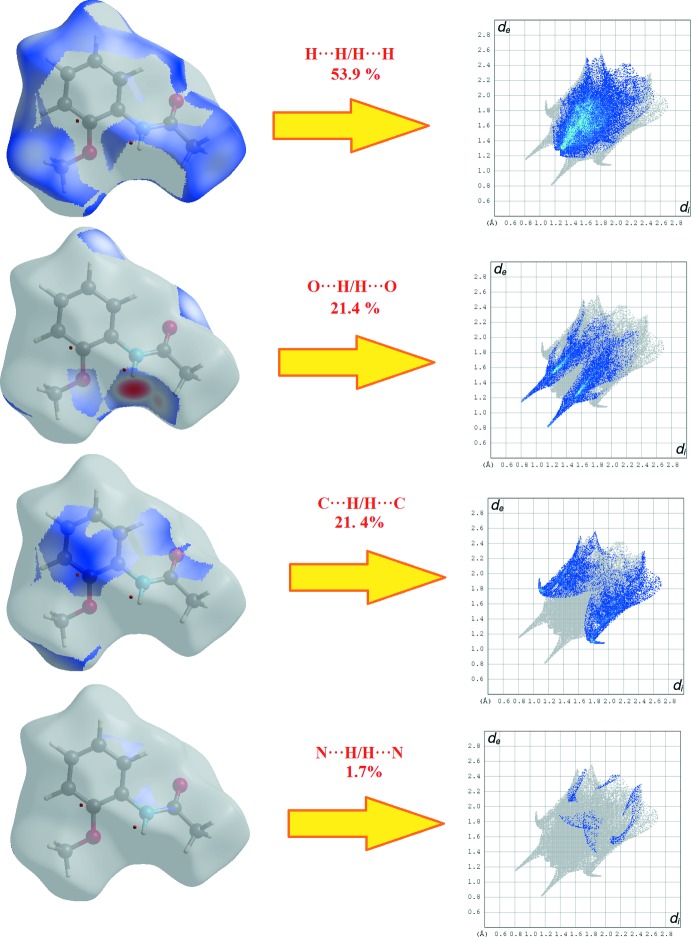
Two-dimensional fingerprint plots with a *d*_norm_ view of the H⋯H/H⋯H (53.9%), C⋯H/H⋯C (21.4%), O⋯H/H⋯O (21.4%) and N⋯H/ H⋯N (1.7%) contacts in the title compound.

**Figure 6:**
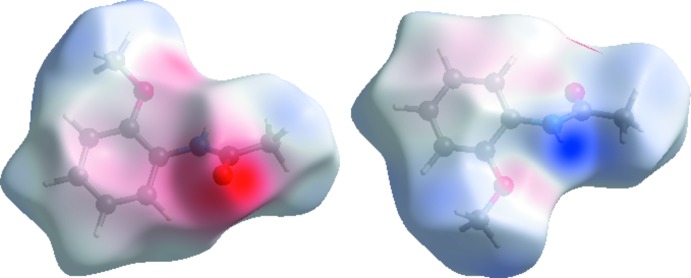
The view of the three-dimensional Hirshfeld surface of the title compound plotted over the electrostatic potentials.

Hirshfeld surface analysis (Spackman & Jayatilaka, 2009 ▸) and the associated two-dimensional fingerprint plots (McKinnon *et al.*, 2007 ▸) were generated using *CrystalExplorer17* (Turner *et al.*, 2017 ▸). Plots of the Hirshfeld surface mapped over *d*_norm_, *d*_i_ and *d*_e_ using a fixed colour scale of −0.5051 (red) to 1.2978 (blue) a.u. are shown in Fig. 3 ▸.. The red spots in the *d*_norm_ plot indicate the inter­molecular contacts associated with the strong hydrogen bonds and inter­atomic contacts such as N—H⋯O. Fig. 4 ▸ shows the *d_norm_* mapped on the Hirshfeld surface to visualize the inter­molecular inter­actions of the title compound. The fingerprint plots complement the Hirshfeld surface, qu­anti­tatively summarizing the nature and type of the inter­molecular contacts by illustrating atom_inside_/atom_outside_ inter­actions (Fig. 5 ▸). The contribution from the H⋯H contacts is observed to be highest towards the Hirshfeld surface with a 53.9% contribution. The contribution from the C—H⋯O hydrogen bond (21.4% contribution) appears as a pair of sharp spikes at *d*_e_ + *d*_i_ =1.9 Å. A view of the three-dimensional Hirshfeld surface plotted over electrostatic potentials in the range −0.1028 to 0.1158 a.u. is shown in Fig. 6 ▸. The hydrogen-bond donors and acceptors are showed as blue and red regions around the atoms corresponding to positive and negative potentials, respectively.

## Database survey   

A search in the Cambridge Structural Database (CSD version 5.39, update of August 2018; Groom *et al.*, 2016 ▸) for *N*-(2-meth­oxy­phen­yl)acetamide derivatives found several similar structures: 3-hy­droxy-7,8-di­meth­oxy­quinolin-2(1*H*)-one (BIZGAT; Song *et al.*, 2008 ▸), 1-(2-meth­oxy­phen­yl)-1*H*-pyrrole-2,5-dione (XEBZIP; Sirajuddin *et al.*, 2012 ▸) and *cis*-cyclo­hexane-1,2-carb­oxy­lic anhydride with *o*- and *p*-anisidine and *m*- and *p*-amino­benzoic acids (BECVAI; Smith *et al.*, 2012 ▸). In the structure of BIZGAT, the mol­ecules are linked into chains by N—H⋯O hydrogen bonds as in the title structure.

## Synthesis and crystallization   

This compound was formed as by-product in the synthesis of a benzamide derivative from the reaction between an oxazolone with *o*- meth­oxy­aniline (Samad & Hawaiz, 2019 ▸) in the presence of acetic acid as solvent. The reaction mixture was refluxed for 2 h, cooled, poured into water, filtered and dried. The remaining filtrate was left for seven days to obtain good-quality crystals.

## Refinement   

Crystal data, data collection and structure refinement details are summarized in Table 2 ▸. The H atoms were positioned geometrically and refined using a riding model with C—H = 0.93 Å for aromatic H atoms, C—H = 0.96 Å for methyl H atoms, and with *U*_iso_(H) = 1.2–1.5 U_eq_(C).

## Supplementary Material

Crystal structure: contains datablock(s) I. DOI: 10.1107/S2056989019006972/mw2145sup1.cif

Structure factors: contains datablock(s) I. DOI: 10.1107/S2056989019006972/mw2145Isup2.hkl

CCDC reference: 1899995

Additional supporting information:  crystallographic information; 3D view; checkCIF report

## Figures and Tables

**Table 1:** Hydrogen-bond geometry (Å, °) *Cg*1 is the centroid of the C3–C8 ring.

*D*—H⋯*A*	*D*—H	H⋯*A*	*D*⋯*A*	*D*—H⋯*A*
N1—H1⋯O2^i^	0.86	2.10	2.9486 (17)	168
C1—H1*B*⋯O2^i^	0.96	2.56	3.378 (2)	143
C1—H9*B*⋯*Cg*1^ii^	0.96	2.61	3.387	139

Symmetry codes: (i) 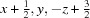; (ii) 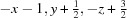.

**Table 2:** Experimental details

Crystal data	
Chemical formula	C_9_H_11_NO_2_
*M*_r_	165.19
Crystal system, space group	Orthorhombic, *P* *b* *c* *a*
Temperature (K)	296
*a*, *b*, *c* (Å)	9.5115 (7), 18.7385 (19), 10.0216 (8)
*V* (Å^3^)	1786.2 (3)
*Z*	8
Radiation type	Mo *K*α
μ (mm^−1^)	0.09
Crystal size (mm)	0.43 × 0.39 × 0.37

Data collection	
Diffractometer	Stoe IPDS 2
Absorption correction	Integration (*X-RED32*; Stoe & Cie, 2002 ▸)
*T*_min_, *T*_max_	0.946, 0.978
No. of measured, independent and observed [*I* > 2σ(*I*)] reflections	14575, 1748, 1168
*R*_int_	0.090
(sin θ/λ)_max_ (Å^−1^)	0.617

Refinement	
*R*[*F* ^2^ > 2σ(*F* ^2^)], *wR*(*F* ^2^), *S*	0.050, 0.148, 1.05
No. of reflections	1748
No. of parameters	111
H-atom treatment	H-atom parameters constrained
Δρ_max_, Δρ_min_ (e Å^−3^)	0.13, −0.12

Computer programs: *X-AREA* and *X-RED32* (Stoe & Cie, 2002 ▸), *SHELXT2018* (Sheldrick, 2015*a* ▸), *SHELXL2018* (Sheldrick, 2015*b* ▸), *ORTEP-3 for Windows* and *WinGX* (Farrugia, 2012 ▸), *Mercury* (Macrae *et al.*, 2006 ▸) and *PLATON* (Spek, 2009 ▸).

## supplementary crystallographic information

### Crystal data

**Table:**

C_9_H_11_NO_2_	*D*_x_ = 1.229 Mg m^−^^3^
*M**_r_* = 165.19	Mo *K*α radiation, λ = 0.71073 Å
Orthorhombic, *P**b**c**a*	Cell parameters from 26458 reflections
*a* = 9.5115 (7) Å	θ = 2.0–28.3°
*b* = 18.7385 (19) Å	µ = 0.09 mm^−^^1^
*c* = 10.0216 (8) Å	*T* = 296 K
*V* = 1786.2 (3) Å^3^	Prism, yellow
*Z* = 8	0.43 × 0.39 × 0.37 mm
*F*(000) = 704	

### Data collection

**Table:**

Stoe IPDS 2 diffractometer	1748 independent reflections
Radiation source: sealed X-ray tube, 12 x 0.4 mm long-fine focus	1168 reflections with *I* > 2σ(*I*)
Plane graphite monochromator	*R*_int_ = 0.090
Detector resolution: 6.67 pixels mm^-1^	θ_max_ = 26.0°, θ_min_ = 2.2°
rotation method scans	*h* = −11→10
Absorption correction: integration (X-RED32; Stoe & Cie, 2002)	*k* = −22→22
*T*_min_ = 0.946, *T*_max_ = 0.978	*l* = −12→12
14575 measured reflections	

### Refinement

**Table:**

Refinement on *F*^2^	0 restraints
Least-squares matrix: full	Hydrogen site location: inferred from neighbouring sites
*R*[*F*^2^ > 2σ(*F*^2^)] = 0.050	H-atom parameters constrained
*wR*(*F*^2^) = 0.148	*w* = 1/[σ^2^(*F*_o_^2^) + (0.0718*P*)^2^] where *P* = (*F*_o_^2^ + 2*F*_c_^2^)/3
*S* = 1.05	(Δ/σ)_max_ < 0.001
1748 reflections	Δρ_max_ = 0.13 e Å^−^^3^
111 parameters	Δρ_min_ = −0.12 e Å^−^^3^

### Special details

**Table:**

Geometry. All esds (except the esd in the dihedral angle between two l.s. planes) are estimated using the full covariance matrix. The cell esds are taken into account individually in the estimation of esds in distances, angles and torsion angles; correlations between esds in cell parameters are only used when they are defined by crystal symmetry. An approximate (isotropic) treatment of cell esds is used for estimating esds involving l.s. planes.

### Fractional atomic coordinates and isotropic or equivalent isotropic displacement parameters (Å^2^)

**Table:**

	*x*	*y*	*z*	*U*_iso_*/*U*_eq_	
O1	0.64879 (13)	0.70439 (8)	0.55583 (15)	0.0835 (5)	
O2	0.25840 (10)	0.58665 (10)	0.69551 (15)	0.0943 (6)	
N1	0.49079 (12)	0.60150 (8)	0.65857 (16)	0.0654 (5)	
H1	0.572027	0.603270	0.696112	0.078*	
C3	0.48608 (15)	0.61300 (10)	0.5196 (2)	0.0622 (5)	
C8	0.57097 (16)	0.66649 (11)	0.4655 (2)	0.0678 (5)	
C2	0.37959 (16)	0.58800 (10)	0.7378 (2)	0.0702 (5)	
C4	0.40284 (18)	0.57277 (11)	0.4362 (2)	0.0739 (6)	
H4	0.346101	0.537006	0.471444	0.089*	
C7	0.5712 (2)	0.67836 (14)	0.3299 (2)	0.0872 (7)	
H7	0.627687	0.713842	0.293428	0.105*	
C1	0.4131 (2)	0.57364 (14)	0.8795 (2)	0.0937 (8)	
H1A	0.349072	0.599462	0.935617	0.141*	
H1B	0.507637	0.588706	0.897970	0.141*	
H1C	0.404506	0.523450	0.896863	0.141*	
C5	0.4032 (2)	0.58533 (13)	0.3000 (3)	0.0917 (7)	
H5	0.346474	0.558364	0.243764	0.110*	
C6	0.4874 (2)	0.63744 (16)	0.2492 (3)	0.0996 (8)	
H6	0.487972	0.645447	0.157579	0.119*	
C9	0.7372 (2)	0.75987 (14)	0.5065 (3)	0.1092 (9)	
H9A	0.802856	0.740327	0.443554	0.164*	
H9B	0.787667	0.781102	0.579383	0.164*	
H9C	0.680779	0.795524	0.463403	0.164*	

### Atomic displacement parameters (Å^2^)

**Table:**

	*U*^11^	*U*^22^	*U*^33^	*U*^12^	*U*^13^	*U*^23^
O1	0.0703 (8)	0.0959 (10)	0.0843 (11)	−0.0264 (7)	0.0062 (7)	−0.0008 (8)
O2	0.0401 (6)	0.1531 (15)	0.0896 (11)	−0.0056 (7)	0.0017 (6)	0.0087 (10)
N1	0.0397 (6)	0.0882 (11)	0.0682 (11)	−0.0052 (6)	−0.0012 (6)	0.0051 (8)
C3	0.0445 (7)	0.0737 (11)	0.0683 (13)	0.0031 (7)	0.0007 (7)	−0.0008 (9)
C8	0.0530 (8)	0.0804 (12)	0.0699 (14)	0.0004 (9)	0.0043 (8)	0.0014 (10)
C2	0.0459 (8)	0.0903 (14)	0.0744 (14)	−0.0029 (9)	0.0031 (8)	0.0061 (11)
C4	0.0579 (9)	0.0824 (13)	0.0813 (16)	−0.0002 (9)	−0.0059 (9)	−0.0065 (11)
C7	0.0767 (13)	0.1117 (18)	0.0732 (17)	0.0005 (12)	0.0110 (11)	0.0104 (13)
C1	0.0611 (11)	0.142 (2)	0.0778 (16)	−0.0005 (12)	0.0054 (10)	0.0168 (14)
C5	0.0773 (12)	0.1154 (19)	0.0823 (17)	0.0060 (13)	−0.0135 (12)	−0.0179 (14)
C6	0.0955 (16)	0.134 (2)	0.0697 (16)	0.0068 (15)	0.0001 (12)	0.0016 (16)
C9	0.0960 (15)	0.1067 (18)	0.125 (2)	−0.0392 (14)	0.0343 (15)	−0.0085 (17)

### Geometric parameters (Å, º)

**Table:**

O1—C8	1.368 (2)	C7—C6	1.370 (3)
O1—C9	1.426 (2)	C7—H7	0.9300
O2—C2	1.2285 (19)	C1—H1A	0.9600
N1—C2	1.347 (2)	C1—H1B	0.9600
N1—C3	1.410 (2)	C1—H1C	0.9600
N1—H1	0.8600	C5—C6	1.362 (3)
C3—C4	1.376 (3)	C5—H5	0.9300
C3—C8	1.397 (3)	C6—H6	0.9300
C8—C7	1.377 (3)	C9—H9A	0.9600
C2—C1	1.480 (3)	C9—H9B	0.9600
C4—C5	1.385 (3)	C9—H9C	0.9600
C4—H4	0.9300		
			
C8—O1—C9	117.93 (18)	C2—C1—H1A	109.5
C2—N1—C3	125.90 (14)	C2—C1—H1B	109.5
C2—N1—H1	117.1	H1A—C1—H1B	109.5
C3—N1—H1	117.1	C2—C1—H1C	109.5
C4—C3—C8	119.4 (2)	H1A—C1—H1C	109.5
C4—C3—N1	122.29 (17)	H1B—C1—H1C	109.5
C8—C3—N1	118.33 (16)	C6—C5—C4	119.5 (2)
O1—C8—C7	124.65 (18)	C6—C5—H5	120.2
O1—C8—C3	115.38 (18)	C4—C5—H5	120.2
C7—C8—C3	119.97 (19)	C5—C6—C7	121.5 (2)
O2—C2—N1	122.48 (19)	C5—C6—H6	119.3
O2—C2—C1	122.00 (16)	C7—C6—H6	119.3
N1—C2—C1	115.51 (15)	O1—C9—H9A	109.5
C3—C4—C5	120.2 (2)	O1—C9—H9B	109.5
C3—C4—H4	119.9	H9A—C9—H9B	109.5
C5—C4—H4	119.9	O1—C9—H9C	109.5
C6—C7—C8	119.5 (2)	H9A—C9—H9C	109.5
C6—C7—H7	120.3	H9B—C9—H9C	109.5
C8—C7—H7	120.3		
			
C2—N1—C3—C4	−41.9 (3)	C3—N1—C2—C1	176.54 (19)
C2—N1—C3—C8	139.18 (19)	C8—C3—C4—C5	−0.1 (3)
C9—O1—C8—C7	−0.4 (3)	N1—C3—C4—C5	−179.04 (17)
C9—O1—C8—C3	−179.83 (17)	O1—C8—C7—C6	−179.23 (19)
C4—C3—C8—O1	179.21 (15)	C3—C8—C7—C6	0.2 (3)
N1—C3—C8—O1	−1.8 (2)	C3—C4—C5—C6	0.5 (3)
C4—C3—C8—C7	−0.2 (3)	C4—C5—C6—C7	−0.6 (3)
N1—C3—C8—C7	178.77 (17)	C8—C7—C6—C5	0.2 (4)
C3—N1—C2—O2	−2.5 (3)		

### Hydrogen-bond geometry (Å, º)

*Cg*1 is the centroid of the C3–C8 ring.

**Table:**

*D*—H···*A*	*D*—H	H···*A*	*D*···*A*	*D*—H···*A*
N1—H1···O2^i^	0.86	2.10	2.9486 (17)	168
C1—H1*B*···O2^i^	0.96	2.56	3.378 (2)	143
C1—H9*B*···*Cg*1^ii^	0.96	2.61	3.387	139

Symmetry codes: (i) *x*+1/2, *y*, −*z*+3/2; (ii) −*x*−1, *y*+1/2, −*z*+3/2.
